# Contemporary Series of Robotic-Assisted Distal Ureteral Reconstruction Utilizing Side Docking Position

**DOI:** 10.1590/S1677-5538.IBJU.2014.0601

**Published:** 2015

**Authors:** Rick C. Slater, Nicholas J. Farber, Julie M. Riley, Yaniv Shilo, Michael C. Ost

**Affiliations:** 1 Department of Urology, University of Pittsburgh Medical Center, Pittsburgh, PA, USA; 2 Division of Urology, Rutgers Robert Wood Johnson Medical School, New Brunswick, NJ, USA; 3 Division of Urology, University of New Mexico, Albuquerque, NM, USA

**Keywords:** Video-Assisted Surgery, Ureter, Reconstructive Surgical Procedures

## Abstract

**Purpose:**

The robot-assisted approach to distal ureteral reconstruction is increasingly utilized. Traditionally, the robot is docked between the legs in lithotomy position resulting in limited bladder access for stent placement. We examined the use of side docking of the daVinci robot® to perform distal ureteral reconstruction.

**Materials and Methods:**

A retrospective review of distal ureteral reconstruction (ureteral reimplantation and uretero-ureterostomy) executed robotically was performed at a single institution by a single surgeon. The daVinci robotic® Si surgical platform was positioned at the right side of the patient facing towards the head of the patient, i.e. side docking.

**Results:**

A total of 14 cases were identified from 2011–2013. Nine patients underwent ureteral reimplantation for ureteral injury, two for vesicoureteral reflux, one for ureteral stricture, and one for megaureter. One patient had an uretero-ureterostomy for a distal stricture. Three patients required a Boari flap due to extensive ureteral injury. Mean operative time was 286 minutes (189–364), mean estimated blood loss was 40cc (10–200), and mean length of stay was 2.3 days (1–4). Follow-up renal ultrasound was available for review in 10/14 patients and revealed no long-term complications in any patient. Mean follow-up was 20.7 months (0.1–59.3).

**Conclusion:**

Robot-assisted laparoscopic distal ureteral reconstruction is safe and effective. Side docking of the robot allows ready access to the perineum and acceptable placement of the robot to successfully complete ureteral repair.

## INTRODUCTION

Laparoscopic techniques for ureteral reimplantation and reconstruction continue to grow. The technique and efficacy of the laparoscopic ureteral reimplantation has been well described (1–3). However, creating a non-refluxing ureteral reimplantation laparoscopically is technically very difficult, and has translated into poor adoption of the technique. With the introduction of the daVinci Surgical System® (Intuitive Surgical, Sunnyvale, CA) minimally invasive surgery has now allowed surgeons to accomplish increasingly complex procedures with a shorter learning curve and better efficacy (4).

Classically, the position of a robotic-assisted laparoscopic ureteral reimplantation is described by placing the patient in lithotomy position followed by steep Trendelenberg position and then the robot is docked between the patient's legs. This position, however, results in limited access to the bladder for retrograde placement of a ureteral stent. Previously, side docking of the daVinci® robot has been described for various gynecologic surgeries (5, 6) as well as for performing a radical prostatectomy (7). We present an alternative docking position which simplifies surgical set-up, allows ready access to the bladder for stent placement and may ultimately lead to shorter operating time without compromising surgical technique.

## MATERIALS AND METHODS

Retrospective chart review was performed on all patients of the senior author's who underwent robotic assisted laparoscopic ureteral reconstruction (i.e. ureteral reimplantation and uretero-ureterostomy) utilizing a side docking position.

Preoperatively, all patients were evaluated with retrograde pyelograms, except those with vesicoureteral reflux (VUR) who were imaged with voiding cystourethrograms. Preoperative management included ureteral stenting or nephrostomy tube placement for patients with ureteral injury or stricture, and observation or deflux in patients with VUR. Routine preoperative labs, including serum creatinine and urinalysis, were obtained in all patients.

**Figure 1:**
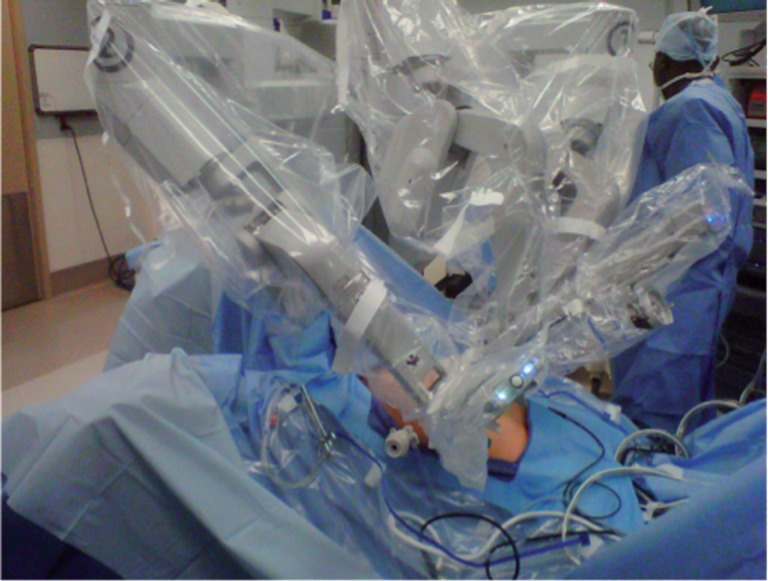
daVinci robotic Si surgical platform port placement.

**Figure 2:**
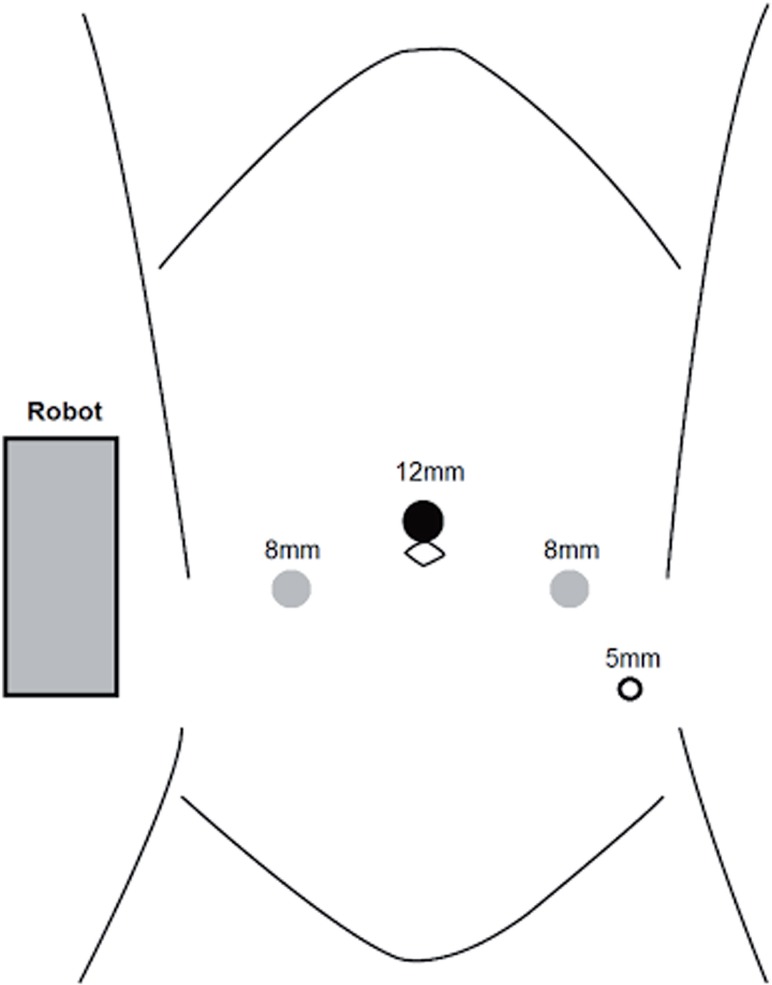
Side docked position.

All operations employed the daVinci robotic Si surgical platform® (Intuitive Surgical, Sunnyvale, CA), with the robot docked on the patient's right side parallel to the operative Table, e.g. “side docked” (Figures 1 and 2) (8). The patient was positioned in dorsal lithotomy position atop a memory foam pad to resist sliding, and legs were placed in yellow fin stirrups. The patient was then placed in a Trendelenberg position. The trocar placement did not differ significantly from traditional docking positions; we utilized an umbilical port for camera placement and the robotic ports were placed 8 to 10cm apart and triangulated about the camera port with adjustments made to avoid the anterior superior iliac spine.

Ureteral reimplant performed for VUR utilized the non-refluxing Lich Gregoire method (9). The ureter was identified and dissected towards the bladder until its attachment to the bladder was visualized. The detrusor muscle was divided from mucosa. A suture was used to advance the ureteral orifice caudally towards the bladder neck. The ureter was then tunneled atop the bladder mucosa and the muscle closed atop the ureter. Ureteral reimplant executed for ureteral injury, stricture, or megaureter employed the Le Duc technique (10). The ureter was mobilized and introduced into the bladder through a short transmural channel in a nonrefluxing fashion. Distally, the ureteral end was widely spatulated and resulted in a distal ureteral plate that was fixed to the bladder mucosa, while the non-spatulated ureter remained unfixed. In all patients, ureteral stents and urethral Foley catheter were placed in a retrograde fashion during the procedure and Jackson-Pratt drains placed at the end of the operation.

Patients were postoperatively evaluated in the office setting approximately three to six weeks after the operation, with cystoscopy and stent removal. Additional follow-up with in-office renal ultrasound was scheduled at three months after surgery and yearly thereafter to assess the repair.

We collected the following demographic and procedural data from the electronic medical records of all patients: age, gender, body mass index (BMI), American Society of Anesthesiologists (ASA) score, estimated blood loss (EBL), indication for surgery, operative time, time from ureteral injury, laterality of the operation, and intraoperative complications. Operative time was defined as time from start of incision to cessation of anesthesia.

Early postoperative outcomes were also extracted from the electronic medical record, including hospital length of stay (LOS). Postoperative complications were defined using the Clavien grading system (11). Office notes were reviewed for results of in-office renal ultrasound. We assessed change in renal function by comparing preoperative and postoperative serum creatinine (sCr) using a paired-sample Student t test. Statistical significance was defined as p<0.05.

## RESULTS

From March 2011 to September 2013 a total of 14 patients (13 female, 1 male) with a mean age of 39 years were identified and included in the study group. Indications for the procedure included ureteral injury during primary hysterectomy in 9 patients, vesicoureteral reflux in 2 patients, congenital stricture in 2 patients and megaureter in 1 patient. Demographic data is listed in Table-1.

**Table 1:** Patient Demographic Data and Operative Data.

Patient	Age, years*	Side	Indication	Preop. Management	Procedure	OT	Postop. Complications	Postop. Imaging	FU
1	45	R	HI	NT	UR	288	None	N	4
2	34	R	MU	Observation	UR, MT	322	None	N	7
3	41	R	HI	Stent	UR, UL	364	None	Y	20
4	37	R	HI	NT	UR, UL	236	None	Y	6
5	22	L	VUR	Observation	UR	241	None	Y	59
6	21	R	VUR	Deflux	UR	224	None	Y	40
7	49	B	HI	NT	UR, UL	362	None	Y	24
8	61	R	HI	NT	UR	350	None	Y	44
9	47	R	HI	Stent	UR, BF	366	None	Y	26
10	28	L	Stricture	Stent	UU	189	None	Y	23
11	37	L	HI	Stent	UR, UL, BF	328	Fever	Y	12
12	64	L	Stricture	Stent	UR, UL, BF	251	None	Y	24
13	35	L	HI	NT	UR	235	None	N	0
14	25	R	HI	NT	UR	254	None	N	0

* Data include age (years); side of reconstruction (**R =** right; L = left; **B =** bilateral); indication for ureteral reconstruction (**HI =** Hysterectomy injury; **MU =** megaureter; **VUR =** vesicoureteral reflux), preoperative management (**NT =** nephrostomy tube), operative procedure (**UR =** ureteral reimplantation; **MR =** megaureter tapering; **UL =** ureterolysis; **UU =** ureteroureterostomy; **BF =** Boari Flap), operative time (**OT =** minutes), postoperative complications, postoperative imaging (**Y =** yes; **N =** No), and duration of follow-up (FU = months)

Operative and postoperative data are listed in Tables 1 and 2. Mean operative time was 286 minutes (189–364 minutes) and mean EBL was 40.0cc (10–200cc). All the procedures were completed by the side-docking method without the need for re-docking. There was one intraoperative complication: a contralateral ureter was erroneously reimplanted and required reoperation and reimplantation of the correct ureter. All surgeries were completed without conversion to open or the need for re-docking. There was a single postoperative Clavien grade I complication (postoperative fever).

**Table 2:** Preoperative, Operative and Postoperative Data.

Patient Variables		
Age (years)		39.0±13.3
Female gender, n (%)		13(93)
BMI (Kg/m2)		26.9±7.8
ASA Score		2.1±0.3
**Preop. sCr (mg/dL)**		
	Mean	0.9±0.2
	Range	0.6–1.2
**Operative variables**		
	Mean Estimated blood loss (cc)	40 (10–200)
	Mean Operative time (min)	286 (189–364)
	Intraoperative complications, n (%)	1 (7.2)
**Postoperative variables**		
	Hospital stay (days)	2.3 (1.0–4.0)
**Postop. Complications, n (%)**		
	Grade I-II	1 (7.2)
	Grade III-V	0 (0)
	Postop. Transfusion, n (%)	0 (0)
**Postop. sCr nadir (mg/dL)**		
	Mean	0.9±0.2
	Range	0.5–1.3
Mean time to stent removal (days)		49 (26–82)
Mean follow-up (months)		20.7 (0.1–59.3)

*sCr = serum creatinine; **BMI =** body mass index; **AsA =** American Society of Anesthesiologists; **sCr =** serum creatinine

Mean length of stay was 2.3 days (1–4 days). Creatinine was available for analysis in 12 of 14 patients. The difference between preoperative and postoperative sCr was not statistically significant (p=68). Follow-up renal ultrasound was available for review in 10 out of 14 patients and demonstrated no evidence of complications in any patient.

## DISCUSSION

Ureteral reconstruction can be accomplished using a variety of open procedures and has been described in the urologic literature with excellent long-term outcomes. However, open surgery is associated with more blood loss, postoperative pain, and longer lengths of hospital stay (2). The introduction of the daVinci robotic system® has changed the landscape of minimally invasive surgery. Despite the higher operating costs, longer setup, and loss of tactile feedback of the current robotic system, the benefits of a three-dimensional field of vision, increased degrees of freedom of movement, tremor elimination, and motion scaling make robotic ureteral reconstruction advantageous (12, 13).

Surgical literature has previously reported successful use of robotic side-docking for pelvic procedures, namely for obstetrics and gynecology operations (5, 6). Two urologic series of robotic side docking have been published in the literature, but their cohorts consisted of primarily urologic oncology cases and included only a single patient with ureteral reconstruction (8, 14). We present, to our knowledge, the first series of patients undergoing robotic assisted ureteral reconstruction with a side docking position.

Our results suggest several important findings. First, our case series demonstrates that side docking of the robot is comparable in operative time to other studies using the conventional docking approach (15, 16). Specifically, our mean operative time was 286.4 minutes which is similar to the 221 minutes reported in the largest case series of conventionally docked robotic distal ureteral reconstructions (16). Of note, our operative times include the entire duration of surgery, not just robotic console time, and may account for some of the disparity between our operative time and the literature time. In addition, many of our patients had additional concurrent procedures performed (e.g. ureterolysis and Boari flap) and nearly all had previously undergone abdominal surgery with subsequent formation of adhesions, both of which prolong operative times. Our mean length of stay of 2.3 days was also similar to the literature means of 1.6 to 2.5 days using conventional docking, while our mean EBL of 40cc was also on par with means of 50cc to 171cc quoted in the literature (16, 17). Our single postoperative complication a Clavien I postoperative fever–and the absence of any long-term complications (as assessed by ultrasound and office evaluation) demonstrates the short and long-term safety of the repair. Additionally, the side-docking of the robot affords the safety advantage of requiring less abduction of the patient's legs, as the robot is no longer in that potential space. No patients in our series suffered from peroneal nerve injury or any musculoskeletal positioning complications. In patients with a history of hip surgery or muscle contractures, side-docking of the robot is an excellent, safe alternative to the traditional docking approach. Overall, our results suggest that the side-docking approach is safe, effective, and comparable to the conventional docking approach.

Robotic ureteral reconstruction with intracorporeal double J ureteral stent placement has been well described but poses several challenges (18, 19). First, confirming stent placement intracorporeally is often difficult and stent migration has been described as a complication (20). In our series, we had no stent migration, which may be attributed to the excellent visualization appreciated in typical ureteral stent placement. The direct access to the lithotomy position afforded by the side-docking position gives the assistant the opportunity to place a stent using a rigid rather than flexible cystoscope and therefore a better field of vision. The ability to easily place retrograde stents also allows for visual confirmation of both the patency of the ureter and a good curl of the distal end of the stent. Another challenge to conventionally docked robotic ureteral reconstruction is that intraoperative stent placement may be cumbersome with the robot blocking urethral access and requiring undocking of the robot or repositioning of the patient. Conversely, side docking of the robot allows easy cystoscopic access to the bladder for retrograde stent placement, especially when the ureter has been completely transected and the injury is managed via a nephrostomy tube.

There are several limitations to this study. First, its retrospective nature and relatively small sample size from a single surgeon introduce a possible selection bias. Nevertheless, this preliminary data may prompt other surgeons to adopt the side-docking approach to ureteral reconstruction and generate additional, larger studies of the approach. Second, we were unable to obtain the same surgeon's data for comparison with the conventional docking approach. Third, postoperative imaging was unavailable in 4/10 patients, making it harder to determine the true success rate of the operation. However, two of those four patients did receive in-office follow-up at 4 and 7 months postoperatively and neither had evidence of complications on evaluation. Moreover, 10/14 patients had extended follow-up with imaging and none had any long-term complications. Finally, this case series had a wrong site intraoperative complication. The error is attributed to the patient's prior abdominal surgery, which caused such extensive fibrosis that the contralateral ureter was shifted to the intended side and was mistaken for the right ureter. The patient's correct ureter was subsequently reimplanted with no short or long-term postoperative complications.

## CONCLUSIONS

Side docking of the robot during robotic assisted laparoscopic ureteral reconstruction of the distal ureter confers some benefit over the conventional docking approach, as the surgeon has unrestricted, ready access to the perineum for retrograde stent placement without undocking the robot or repositioning the patient. This approach is also safe and effective in terms of operative time, length of stay, and EBL comparable to literature values of the conventional docking approach.
